# Reinterpretation of purported molting evidence in the Thermopolis *Archaeopteryx*

**DOI:** 10.1038/s42003-021-02349-x

**Published:** 2021-07-05

**Authors:** Yosef Kiat, Peter Pyle, Amir Balaban, Jingmai K. O’Connor

**Affiliations:** 1 Animal Flight Laboratory, Department of Evolutionary and Environmental Biology and the Institute of Evolution, University of Haifa, Haifa, Israel; 2 The Nili & David Jerusalem Bird Observatory (JBO), Israel Ornithological Center, Society for the Protection of Nature in Israel, Jerusalem, Israel; 3 The Institute for Bird Populations, Petaluma, CA USA; 4 Field Museum of Natural History, Chicago, IL USA

**Keywords:** Palaeontology, Zoology

**Arising from** T.G. Kaye et al. *Communications Biology* 10.1038/s42003-020-01467-2 (2020)

Feather molt is an important process in the avian annual cycle that is affected by several variables, including flight ability, migration strategy, habitat preference, and morphology^1,2^. Understanding molt strategies in feathered non-avian dinosaurs and stem birds has the potential to expand our knowledge of the ecology and flight ability of these extinct species and shed light on the evolution of this important process in birds. Kaye et al.^3^ described purported feather sheaths in the Thermopolis *Archaeopteryx* (WDC-CSG-100^4^) using laser-stimulated fluorescence to amplify what is visible under normal light and ultraviolet-induced fluorescence^4^. They suggested this finding may support a bidirectional sequential molt with inward and outward renewal of the primary feathers from a single center (node), a pattern that rarely occurs in Neoaves (crown birds). Kaye et al. claim this discovery represents the earliest record of sequential molting in a pennaraptoran (150 million years old), and the first record of molt in an avialan^3^.

Modern birds with sequential molts utilize several strategies, including distal molts from one to four nodes, a proximal molt, and bidirectional sequences starting with a middle primary^5,6^. In addition, some species can shed all primaries in short time intervals (synchronous molt) or with no predictable sequence or direction, during which feather replacement may be non-sequential. In order to determine the specific strategy, the generation and relative feather condition (old vs renewed) of the feathers surrounding growing feathers must also be determined. In the Thermopolis *Archaeopteryx*, specifically, the outermost and innermost primaries would have to be old to indicate a bidirectional sequence starting with a mid-primary (Fig. 1a) as suggested by Kaye et al.^3^. If the outermost and innermost primaries are new, a convergent sequence would be indicated, and if the surrounding feathers are a mix of old and new feathers, a non-sequential molt would be indicated (Fig. 1b). However, feather ages and thus sequence are undeterminable in this fossil specimen.

**Fig. 1:**
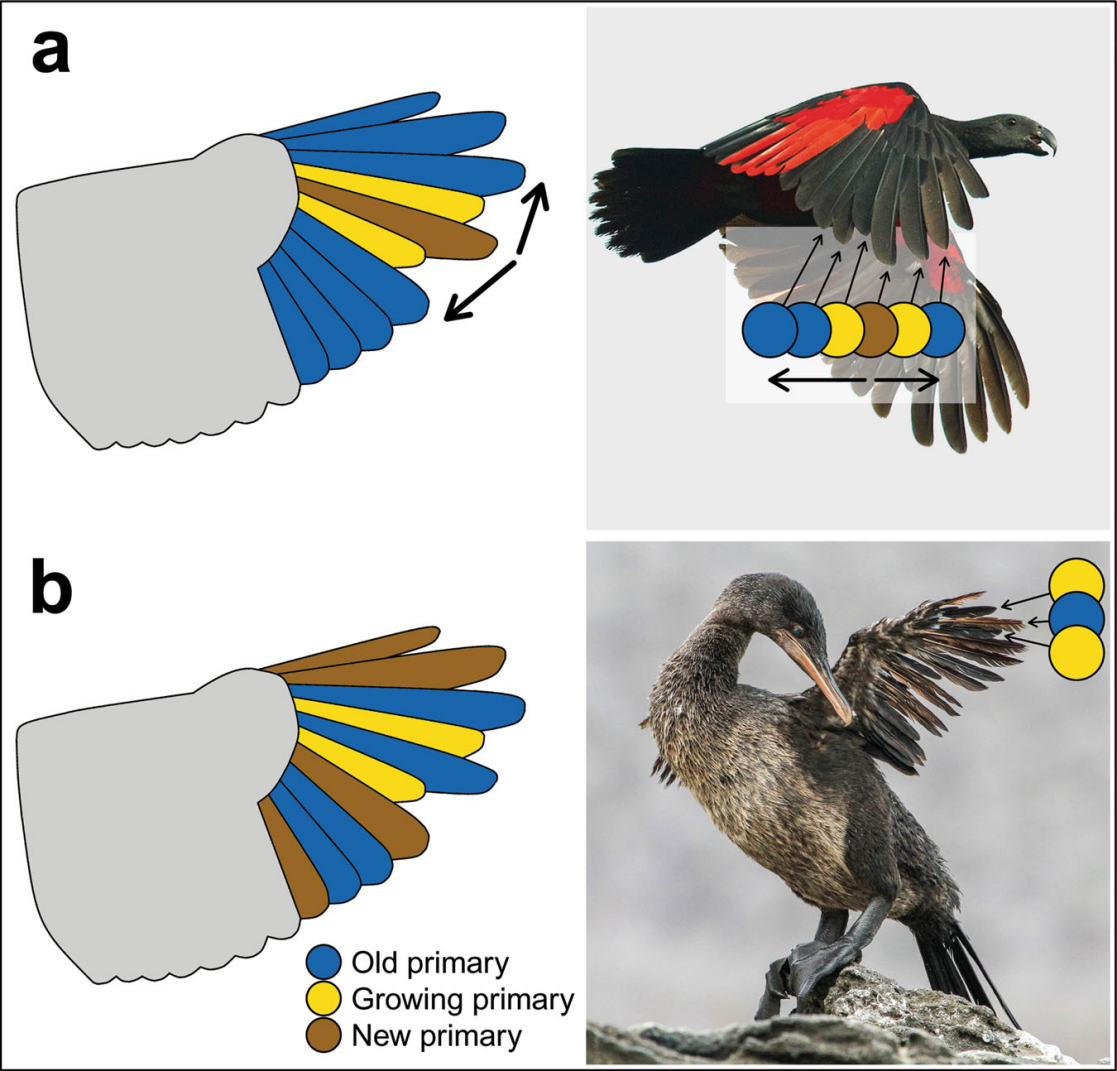
Two optional molt strategies fit a situation of two single feathers in growth in mid-primary feathers. **a** Bidirectional sequence, a sequential molt strategy starting with the molt of a mid-primary and progressing inward and outward. This strategy is rare among modern birds but typical of parrots and falcons, for example, the Pesquet’s Parrot (*Psittrichas fulgidus*; Credit: L. Petersson, ML205975001, The Macaulay Library at the Cornell Lab of Ornithology). **b** Irregular, non-sequential molt, as occurs among flightless birds, for example, the Flightless Cormorant (*Phalacrocorax harrisi*; Credit: L. Martin). This individual demonstrates the possibility of two single molting feathers (probably P5 and P7, P6 is old) when non-sequential molt occurs.

The most common strategy among Neornithes is distal molt from a single node, the innermost primary (P1), as found in many Palaeognathae and Galloanserae species (e.g., *Tinamus*), the clades Strisores and Columbea (the two most likely options to be a sister clade to the rest of the Neoaves), and many other birds including most passerines. This suggests that, among Neoaves, a distal primary molt could be the ancestral molt sequence. While most birds maintain flight performance during the molt by reducing the number of feathers molting within a short time interval^1^, a bidirectional molt sequence allows flight performance to be maintained while a greater number of flight feathers are actively molting within a short time interval^6^. This is an advanced strategy that has evolved in more specialized highly volant birds based on wing morphology and other factors. Specifically, bidirectional molt occurs in parrots (Psittaciformes) and falcons (Falconiformes)^6^, as well as in some species among other advanced clades, e.g., some owls (Strigiformes), some kingfishers (Alcedines), and rarely in Passeriformes^5^. Thus, this strategy is unlikely to have occurred in stem avialans since specialized flight adaptations are unlikely to have emerged at this early stage of avian evolution.

Potential molting and symmetry in missing flight feathers have previously been reported in *Archaeopteryx*^7,8^ and the enantiornithine *Protopteryx*^9^. The London *Archaeopteryx* (BMNH-37001) preserves at least one molting feather^8^. In the Early Cretaceous four-winged volant dromaeosaurid *Microraptor* (IVPP V13352), a sequential molt is evidenced by a succession of three growing primaries^2^. The state of the innermost primary feathers in this specimen could not be ascertained. Thus (contra Kaye et al.^3^), the specific sequential molting strategy in *Microraptor* remains unidentified; both a distal molt sequence (molting from the innermost primary outward) and a bidirectional sequence, as in parrots and falcons (Fig. 1a), are possible. However, owing to the rarity of bidirectional molt strategies among modern birds^6^, a distal molt sequence appears to be more likely for *Microraptor*. Such a strategy is not necessarily plesiomorphic to Paraves; it is also possible that the ancestral paravian molt strategy was non-sequential and that sequential molts coevolved with advanced capabilities for flight^2^.

The conclusion by Kaye et al.^3^ that the fifth (P5) and seventh (P7) primaries on the right-wing in the Thermopolis *Archaeopteryx* are growing is not supported by additional evidence. The P7 tip contour as drawn by the authors (Figure 4 in Kaye et al.^3^) may be incorrect. P7 and P8 may overlap and hide each other as was originally suggested by Mayr et al.^4^ and as appears to occur within the secondaries on the same wing. Furthermore, there is no evidence supporting that P5 is not full length; the graduated lengths of P4 to P6 appear similar to the standard wing morphology shown by other *Archaeopteryx* fossil specimens^4,10^. Hence, we suggest that P5 to P7 are likely full-length feathers or, at best, that the evidence indicating they are actively molting is equivocal. In addition, the orientation of the structures identified as molting feather sheaths^3^ does not match that of the shafts of the same feathers as visible in the specimen or as presented by Mayr et al.^4^. The most noticeable difference is in P5, which Kaye et al. attribute to the first phalanx of the digit II/III^3^, whereas Mayr et al.^4^ attributes this feather to the metacarpal, as also supported by observations from other *Archaeopteryx* specimens^4,10^. Based on the orientation of the feather shafts, the structure identified by Kaye et al. as the molting feather sheath of P5 could instead be the base of P6, whereas the structure identified as the feather sheath of P7 could be that of either P7 or P8. The morphology of the right-wing cannot be accurately evaluated since the contours of the primaries are not clearly preserved.

The position of the preserved feather sheaths identified by Kaye et al.^3^ is also problematic, being located directly adjacent to the bones of the hand. In Neornithes, early Cretaceous enantiornithines^11^, and confuciusornithiforms^12^, this region is occupied by the postpatagium, which has also been identified in the London *Archaeopteryx*^13^. The proximal 9.40% ± 0.02% (mean ± standard deviation; measured from 145 primaries of 13 neoavian species) of each primary, formed by the calamus, is embedded within the postpatagium. If our interpretation that these feathers are full-grown is correct, this suggests that the structures identified by Kaye et al.^3^ are in fact feather calami. If indeed these feathers are growing, the preserved sheath includes portions both outside and inside the postpatagium and thus would still partially represent calami since these two structures (feather sheath and calamus) are continuous^14^. The feather calamus and molting feather sheath are both keratin-based tissue (alpha-keratin or beta-keratin)^14^, and there is no reason why the sheath would be more likely to preserve than the much thicker calamus, nor why the sheath would be reactive under LSF but not the calamus, as claimed by Kaye et al.^3^.

In light of a more comprehensive understanding of neoavian molt strategies the conclusions presented by Kaye et al.^3^ are unsupported; if these primaries are indeed molting feathers, this information alone can neither differentiate between a sequential or non-sequential nor determine the specific direction(s) of a sequential molt pattern. Furthermore, the symmetry described by Kaye et al.^3^ may not inform the molt strategy; even among flightless species, a certain degree of symmetry may coincidentally appear to be present (e.g., Common Ostrich, *Struthio camelus*^15^). We, therefore, suggest that the identification of feather sheaths in the Thermopolis *Archaeopteryx* is equivocal at best, and that these structures are more likely traces of the calami of full-grown feathers, which are normally hidden by wing-coverts.

## Reporting summary

Further information on research design is available in the Nature Research Reporting Summary linked to this article.

## Supplementary information

Reporting Summary

## Data Availability

The data that support the findings of this study are available in https://osf.io/q7xr6/.
